# Supplementary material for: Pyruvate Dehydrogenase Kinase 1 inhibition mediated oxidative phosphorylation enhancement in cartilage promotes osteoarthritis progression
Source: BMC Musculoskelet Disord. 2023 Jul 20;24:597. doi: 10.1186/s12891-023-06585-6 (PMC10357736; doi:10.1186/s12891-023-06585-6)
Supplement: Supplementary file 1 — Additional file 1. [file 12891_2023_6585_MOESM1_ESM.zip › KEGG.Gsea.1653622432464/KEGG_CARDIAC_MUSCLE_CONTRACTION.html]

Details for gene set KEGG\_CARDIAC\_MUSCLE\_CONTRACTION[GSEA]

|  || Dataset | 55457\_collapsed\_to\_symbols.cls.cls #OA\_versus\_normal.cls.cls #OA\_versus\_normal\_repos |
| Phenotype | cls.cls#OA\_versus\_normal\_repos |
| Upregulated in class | OA |
| GeneSet | KEGG\_CARDIAC\_MUSCLE\_CONTRACTION |
| Enrichment Score (ES) | 0.38651678 |
| Normalized Enrichment Score (NES) | 1.6238009 |
| Nominal p-value | 0.0 |
| FDR q-value | 0.03609575 |
| FWER p-Value | 0.465 |
Table: GSEA Results Summary

  

Fig 1: Enrichment plot: KEGG\_CARDIAC\_MUSCLE\_CONTRACTION      
 Profile of the Running ES Score & Positions of GeneSet Members on the Rank Ordered List

  

| SYMBOL | TITLE | RANK IN GENE LIST | RANK METRIC SCORE | RUNNING ES | CORE ENRICHMENT || 1 | UQCR10 | "ubiquinol-cytochrome c reductase, complex III subunit X [Source:HGNC Symbol;Acc:HGNC:30863]" | 53 | 0.987 | 0.0559 | Yes |
| 2 | UQCRC1 | ubiquinol-cytochrome c reductase core protein 1 [Source:HGNC Symbol;Acc:HGNC:12585] | 473 | 0.672 | 0.0641 | Yes |
| 3 | COX5B | cytochrome c oxidase subunit 5B [Source:HGNC Symbol;Acc:HGNC:2269] | 585 | 0.628 | 0.0937 | Yes |
| 4 | COX6A1 | cytochrome c oxidase subunit 6A1 [Source:HGNC Symbol;Acc:HGNC:2277] | 653 | 0.605 | 0.1253 | Yes |
| 5 | RYR2 | ryanodine receptor 2 [Source:HGNC Symbol;Acc:HGNC:10484] | 828 | 0.561 | 0.1458 | Yes |
| 6 | SLC9A6 | solute carrier family 9 member A6 [Source:HGNC Symbol;Acc:HGNC:11079] | 981 | 0.522 | 0.1658 | Yes |
| 7 | UQCRQ | ubiquinol-cytochrome c reductase complex III subunit VII [Source:HGNC Symbol;Acc:HGNC:29594] | 986 | 0.521 | 0.1971 | Yes |
| 8 | COX8A | cytochrome c oxidase subunit 8A [Source:HGNC Symbol;Acc:HGNC:2294] | 1105 | 0.494 | 0.2180 | Yes |
| 9 | ATP1B1 | ATPase Na+/K+ transporting subunit beta 1 [Source:HGNC Symbol;Acc:HGNC:804] | 1177 | 0.481 | 0.2417 | Yes |
| 10 | CACNA2D3 | calcium voltage-gated channel auxiliary subunit alpha2delta 3 [Source:HGNC Symbol;Acc:HGNC:15460] | 1228 | 0.471 | 0.2665 | Yes |
| 11 | CACNA2D2 | calcium voltage-gated channel auxiliary subunit alpha2delta 2 [Source:HGNC Symbol;Acc:HGNC:1400] | 1431 | 0.439 | 0.2774 | Yes |
| 12 | COX7A1 | cytochrome c oxidase subunit 7A1 [Source:HGNC Symbol;Acc:HGNC:2287] | 2045 | 0.356 | 0.2514 | Yes |
| 13 | COX4I1 | cytochrome c oxidase subunit 4I1 [Source:HGNC Symbol;Acc:HGNC:2265] | 2167 | 0.342 | 0.2627 | Yes |
| 14 | ACTC1 | actin alpha cardiac muscle 1 [Source:HGNC Symbol;Acc:HGNC:143] | 2331 | 0.323 | 0.2697 | Yes |
| 15 | MYL2 | myosin light chain 2 [Source:HGNC Symbol;Acc:HGNC:7583] | 2376 | 0.317 | 0.2855 | Yes |
| 16 | TNNC1 | "troponin C1, slow skeletal and cardiac type [Source:HGNC Symbol;Acc:HGNC:11943]" | 2396 | 0.315 | 0.3032 | Yes |
| 17 | COX7A2L | cytochrome c oxidase subunit 7A2 like [Source:HGNC Symbol;Acc:HGNC:2289] | 2517 | 0.302 | 0.3122 | Yes |
| 18 | MYH6 | myosin heavy chain 6 [Source:HGNC Symbol;Acc:HGNC:7576] | 2555 | 0.297 | 0.3274 | Yes |
| 19 | CACNA1F | calcium voltage-gated channel subunit alpha1 F [Source:HGNC Symbol;Acc:HGNC:1393] | 2726 | 0.278 | 0.3310 | Yes |
| 20 | COX6B1 | cytochrome c oxidase subunit 6B1 [Source:HGNC Symbol;Acc:HGNC:2280] | 2915 | 0.259 | 0.3322 | Yes |
| 21 | CACNG4 | calcium voltage-gated channel auxiliary subunit gamma 4 [Source:HGNC Symbol;Acc:HGNC:1408] | 3065 | 0.244 | 0.3354 | Yes |
| 22 | MYH7 | myosin heavy chain 7 [Source:HGNC Symbol;Acc:HGNC:7577] | 3161 | 0.234 | 0.3422 | Yes |
| 23 | COX7A2 | cytochrome c oxidase subunit 7A2 [Source:HGNC Symbol;Acc:HGNC:2288] | 3164 | 0.234 | 0.3563 | Yes |
| 24 | COX7B | cytochrome c oxidase subunit 7B [Source:HGNC Symbol;Acc:HGNC:2291] | 3452 | 0.207 | 0.3465 | Yes |
| 25 | TPM3 | tropomyosin 3 [Source:HGNC Symbol;Acc:HGNC:12012] | 3484 | 0.204 | 0.3565 | Yes |
| 26 | MYL3 | myosin light chain 3 [Source:HGNC Symbol;Acc:HGNC:7584] | 3529 | 0.199 | 0.3652 | Yes |
| 27 | CACNG2 | calcium voltage-gated channel auxiliary subunit gamma 2 [Source:HGNC Symbol;Acc:HGNC:1406] | 3819 | 0.174 | 0.3533 | Yes |
| 28 | UQCRH | ubiquinol-cytochrome c reductase hinge protein [Source:HGNC Symbol;Acc:HGNC:12590] | 3827 | 0.174 | 0.3633 | Yes |
| 29 | UQCRC2 | ubiquinol-cytochrome c reductase core protein 2 [Source:HGNC Symbol;Acc:HGNC:12586] | 4020 | 0.154 | 0.3577 | Yes |
| 30 | CACNA1S | calcium voltage-gated channel subunit alpha1 S [Source:HGNC Symbol;Acc:HGNC:1397] | 4031 | 0.153 | 0.3662 | Yes |
| 31 | COX6C | cytochrome c oxidase subunit 6C [Source:HGNC Symbol;Acc:HGNC:2285] | 4036 | 0.152 | 0.3752 | Yes |
| 32 | FXYD2 | FXYD domain containing ion transport regulator 2 [Source:HGNC Symbol;Acc:HGNC:4026] | 4073 | 0.149 | 0.3814 | Yes |
| 33 | TPM1 | tropomyosin 1 [Source:HGNC Symbol;Acc:HGNC:12010] | 4220 | 0.135 | 0.3783 | Yes |
| 34 | SLC9A1 | solute carrier family 9 member A1 [Source:HGNC Symbol;Acc:HGNC:11071] | 4221 | 0.135 | 0.3865 | Yes |
| 35 | COX7C | cytochrome c oxidase subunit 7C [Source:HGNC Symbol;Acc:HGNC:2292] | 4539 | 0.110 | 0.3685 | No |
| 36 | CACNA2D1 | calcium voltage-gated channel auxiliary subunit alpha2delta 1 [Source:HGNC Symbol;Acc:HGNC:1399] | 4747 | 0.091 | 0.3579 | No |
| 37 | CACNG1 | calcium voltage-gated channel auxiliary subunit gamma 1 [Source:HGNC Symbol;Acc:HGNC:1405] | 4773 | 0.089 | 0.3614 | No |
| 38 | CYC1 | cytochrome c1 [Source:HGNC Symbol;Acc:HGNC:2579] | 4879 | 0.082 | 0.3582 | No |
| 39 | UQCR11 | "ubiquinol-cytochrome c reductase, complex III subunit XI [Source:HGNC Symbol;Acc:HGNC:30862]" | 4917 | 0.078 | 0.3601 | No |
| 40 | UQCRB | ubiquinol-cytochrome c reductase binding protein [Source:HGNC Symbol;Acc:HGNC:12582] | 5003 | 0.073 | 0.3579 | No |
| 41 | COX6A2 | cytochrome c oxidase subunit 6A2 [Source:HGNC Symbol;Acc:HGNC:2279] | 5242 | 0.053 | 0.3426 | No |
| 42 | COX5A | cytochrome c oxidase subunit 5A [Source:HGNC Symbol;Acc:HGNC:2267] | 5329 | 0.045 | 0.3386 | No |
| 43 | ATP1A3 | ATPase Na+/K+ transporting subunit alpha 3 [Source:HGNC Symbol;Acc:HGNC:801] | 5475 | 0.035 | 0.3294 | No |
| 44 | UQCRFS1 | "ubiquinol-cytochrome c reductase, Rieske iron-sulfur polypeptide 1 [Source:HGNC Symbol;Acc:HGNC:12587]" | 5509 | 0.032 | 0.3288 | No |
| 45 | TNNI3 | "troponin I3, cardiac type [Source:HGNC Symbol;Acc:HGNC:11947]" | 5549 | 0.029 | 0.3275 | No |
| 46 | CACNA1C | calcium voltage-gated channel subunit alpha1 C [Source:HGNC Symbol;Acc:HGNC:1390] | 6367 | -0.033 | 0.2659 | No |
| 47 | CACNB3 | calcium voltage-gated channel auxiliary subunit beta 3 [Source:HGNC Symbol;Acc:HGNC:1403] | 6753 | -0.059 | 0.2395 | No |
| 48 | ATP1B4 | ATPase Na+/K+ transporting family member beta 4 [Source:HGNC Symbol;Acc:HGNC:808] | 7306 | -0.103 | 0.2027 | No |
| 49 | TNNT2 | "troponin T2, cardiac type [Source:HGNC Symbol;Acc:HGNC:11949]" | 7441 | -0.114 | 0.1992 | No |
| 50 | CACNB1 | calcium voltage-gated channel auxiliary subunit beta 1 [Source:HGNC Symbol;Acc:HGNC:1401] | 7589 | -0.126 | 0.1955 | No |
| 51 | TPM4 | tropomyosin 4 [Source:HGNC Symbol;Acc:HGNC:12013] | 7974 | -0.153 | 0.1748 | No |
| 52 | TPM2 | tropomyosin 2 [Source:HGNC Symbol;Acc:HGNC:12011] | 8156 | -0.167 | 0.1709 | No |
| 53 | ATP1A2 | ATPase Na+/K+ transporting subunit alpha 2 [Source:HGNC Symbol;Acc:HGNC:800] | 8218 | -0.173 | 0.1767 | No |
| 54 | ATP1A1 | ATPase Na+/K+ transporting subunit alpha 1 [Source:HGNC Symbol;Acc:HGNC:799] | 8282 | -0.177 | 0.1826 | No |
| 55 | CACNG3 | calcium voltage-gated channel auxiliary subunit gamma 3 [Source:HGNC Symbol;Acc:HGNC:1407] | 8677 | -0.207 | 0.1645 | No |
| 56 | ATP2A2 | ATPase sarcoplasmic/endoplasmic reticulum Ca2+ transporting 2 [Source:HGNC Symbol;Acc:HGNC:812] | 9015 | -0.233 | 0.1524 | No |
| 57 | CACNA1D | calcium voltage-gated channel subunit alpha1 D [Source:HGNC Symbol;Acc:HGNC:1391] | 9867 | -0.308 | 0.1049 | No |
| 58 | CACNB2 | calcium voltage-gated channel auxiliary subunit beta 2 [Source:HGNC Symbol;Acc:HGNC:1402] | 9967 | -0.318 | 0.1165 | No |
| 59 | CACNB4 | calcium voltage-gated channel auxiliary subunit beta 4 [Source:HGNC Symbol;Acc:HGNC:1404] | 9992 | -0.320 | 0.1341 | No |
| 60 | ATP1B2 | ATPase Na+/K+ transporting subunit beta 2 [Source:HGNC Symbol;Acc:HGNC:805] | 10595 | -0.383 | 0.1105 | No |
| 61 | ATP1B3 | ATPase Na+/K+ transporting subunit beta 3 [Source:HGNC Symbol;Acc:HGNC:806] | 11424 | -0.490 | 0.0758 | No |
| 62 | SLC8A1 | solute carrier family 8 member A1 [Source:HGNC Symbol;Acc:HGNC:11068] | 12192 | -0.642 | 0.0551 | No |
Table: GSEA details [plain text format]

  

Fig 2: KEGG\_CARDIAC\_MUSCLE\_CONTRACTION      
 Blue-Pink O' Gram in the Space of the Analyzed GeneSet

  

Fig 3: KEGG\_CARDIAC\_MUSCLE\_CONTRACTION: Random ES distribution      
 Gene set null distribution of ES for **KEGG\_CARDIAC\_MUSCLE\_CONTRACTION**
